# Complete mitochondrial genome of *Rhaphidophora duxiu* (Orthoptera: Rhaphidophoridae: Rhaphidophorinae)

**DOI:** 10.1128/mra.00747-24

**Published:** 2025-01-10

**Authors:** Tingting Yu, Yuqing Yao, Xun Bian, Bin Zhang

**Affiliations:** 1 College of Life Sciences and Technology, Inner Mongolia Normal University, Hohhot, China; 2 Guangxi Key Laboratory of Rare and Endangered Animal Ecology, Guangxi Normal University, Guilin, China; University of California Riverside, Riverside, California, USA

**Keywords:** mitogenome, China, Rhaphidophorinae

## Abstract

We present the complete mitochondrial genome of *Rhaphidophora duxiu* from China. The mitogenome of *R. duxiu* is circular, AT-rich (75.3%), and 15,898 bp in length. It comprises 13 protein-coding genes, 2 ribosomal RNA genes, and 22 transfer RNA genes. It is identical in gene content to *Rhaphidophora quadrispina*.

## ANNOUNCEMENT

The rhaphidophorid subfamily Rhaphidophorinae, is a completely wingless taxon in Orthoptera, distributed from South Asia to Australia (1). In China, the subfamily is poorly understood. *Rhaphidophora duxiu* was first reported from Guangxi, China (2). It is a polyphagous species and often appears at night. To contribute to the phylogenomics of Rhaphidophorinae, the complete mitochondrial genome of *R. duxiu* from Guangxi was assembled and annotated.

The specimen of *R. duxiu* analyzed here was collected from Cenwanglaoshan, Tianlin, Guangxi, China (24.4897 N 106.4019 E). The voucher specimen was stored in absolute ethyl alcohol at −4°C in the College of Life Sciences, Guangxi Normal University (GXNU). Total genomic DNA was extracted from the muscle tissues of the hind leg using the TIANamp Genomic DNA Kit (TIANGEN) following the instructions and sent to Beijing Berry Genomics Co., Ltd. for high-throughput sequencing. The 150-base-pair paired-end library was constructed with the MGIEasy Kit (MGI) as well as sequenced on an Illumina NovaSeq 6000 (Illumina Inc.). The raw data were processed with fastp v.0.20.0 (3), by trimming adapters and primers, filtering reads with phred quality <Q5 and filtering reads with N base number >3. The sequencing generated 23,054,251 reads that were filtered. The number of reads listed is after QC. The entire mitochondrial genome sequence was assembled by NOVOPlasty 4.3.5 (4) using type mito, genome range 14,000–18,000, kmer 39, max memory 16, extended log 0, and *Rhaphidophora quadrispina* (NC067624) as reference sequence. A single *R. duxiu* mitochondrial contig with 191× coverage was identified by Quast 5.2.0 using the default settings (5). The mitochondrial genome start position was adjusted to correspond with *Rhaphidophora quadrispina*. The annotation was performed on the MITOS webserver (6). Gene start and stop positions were confirmed by comparison to 33 complete mitochondrial genomes of Rhaphidophoroidea in GenBank and using standard, universally accepted initiation and termination codons as defined in the invertebrate mitochondrial genetic code (7, 8). Nucleotide identities were calculated by BLAST 2.15.0+ search using the default settings (9).

**Fig 1:**
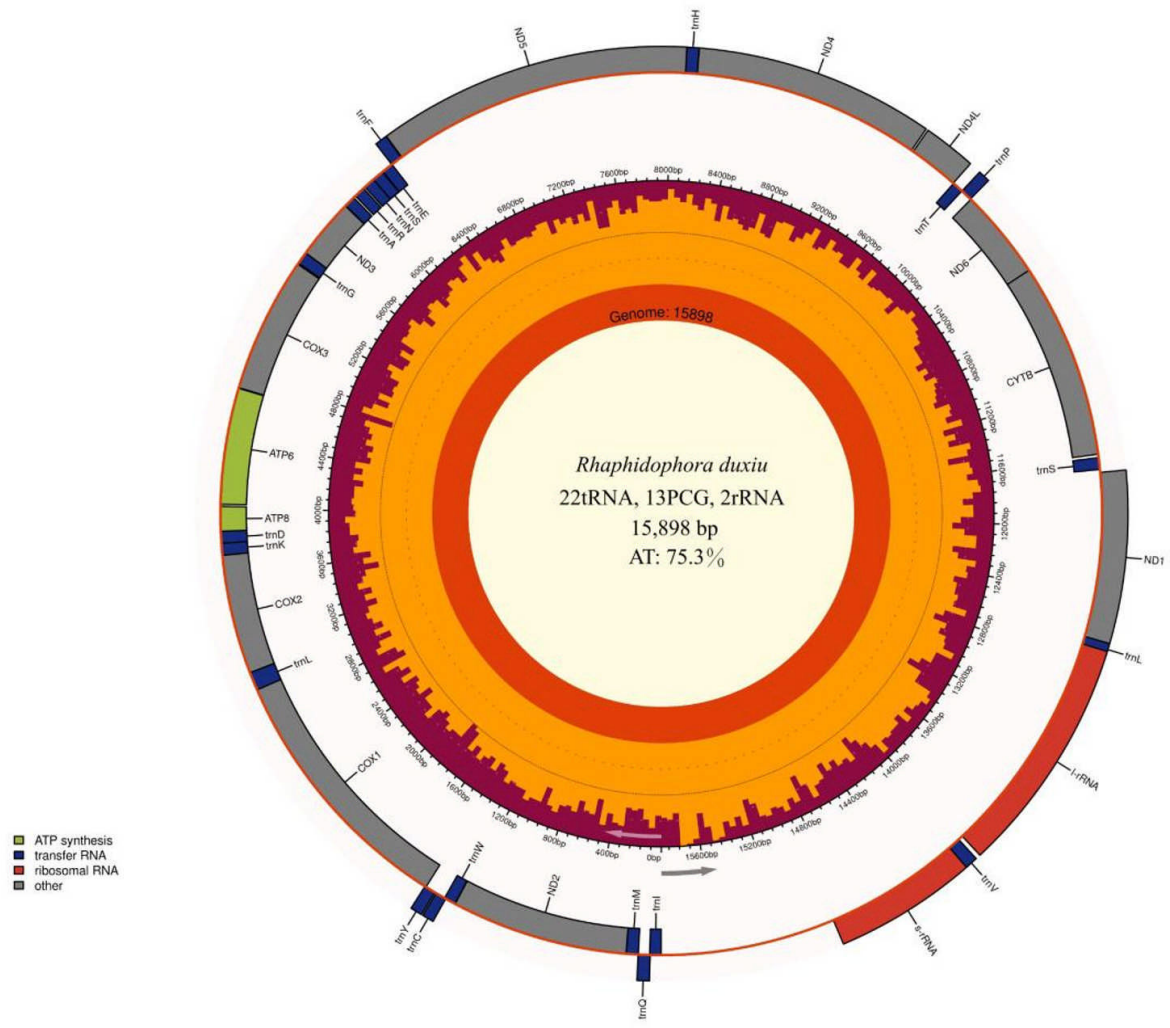
The complete mitochondrial genome map of *Rhaphidophora duxiu*. The genome was annotated using MITOS webserver and mapped with Chloroplot 0.2.4 (12). The innermost ring displays the GC content and direction of transcription, as indicated by the two arrows. The final ring shows the genes. Genes transcribed clockwise are on the inside, while counterclockwise transcriptions are on the outside. The color coding corresponds to genes of different groups as listed in the key in the bottom left.

The complete circular mitochondrial genome of *R. duxiu* is 15,898 bp in length and has an AT bias of 75.3% (GenBank accession number PP953500). The GC content is 24.7%. The complete mitogenome contains 37 genes including 13 protein-coding genes, 2 ribosomal RNA genes, 22 transfer RNA genes, and a control region (D-loop) (Fig. 1). Except ND1 started with TTG, the other protein-coding genes initiate with ATN (Table 1). The TAA termination codon is found in all genes except ND1 (TAG), ATP8, and ND4 (T, stop codon is completed by the addition of 3′ A residues to the mRNA, as is common in animal mitochondrial genomes [10, 11]). The entire mitochondrial genome sequence of *R. duxiu* is 85.86% similar to the genome of *R. quadrispina* from China.

**TABLE 1:** Annotation and gene organization of the *Rhaphidophora duxiu*

Gene	Type	Minimum nucleotide position	Maximum nucleotide position	Length	Start codon	Stop codon	Direction
tRNA-Ile	tRNA	1	67	67	–^*a*^	–	Forward
tRNA-Gln	tRNA	64	133	70	–	–	Reverse
tRNA-Met	tRNA	132	202	71	–	–	Forward
ND2	CDS	203	1,231	1,029	ATG	TAA	Forward
tRNA-Trp	tRNA	1,233	1,301	69	–	–	Forward
tRNA-Cys	tRNA	1,293	1,357	65	–	–	Reverse
tRNA-Tyr	tRNA	1,366	1,432	67	–	–	Reverse
COX1	CDS	1,425	2,966	1,542	ATT	TAA	Forward
tRNA-Leu	tRNA	2,976	3,042	67	–	–	Forward
COX2	CDS	3,043	3,735	693	ATT	TAA	Forward
tRNA-Lys	tRNA	3,736	3,806	71	–	–	Forward
tRNA-Asp	tRNA	3,805	3,873	69	–	–	Forward
ATP8	CDS	3,874	4,017	144	ATA	T	Forward
ATP6	CDS	4,029	4,703	675	ATA	TAA	Forward
COX3	CDS	4,707	5,495	789	ATG	TAA	Forward
tRNA-Gly	tRNA	5,502	5,573	72	–	–	Forward
ND3	CDS	5,574	5,927	354	ATT	TAA	Forward
tRNA-Arg	tRNA	5,930	5,995	66	–	–	Forward
tRNA-Ala	tRNA	6,008	6,072	65	–	–	Forward
tRNA-Asn	tRNA	6,083	6,149	67	––	–	Forward
tRNA-Ser	tRNA	6,149	6,216	68	–	–	Forward
tRNA-Glu	tRNA	6,219	6,286	68	–	–	Forward
tRNA-Phe	tRNA	6,284	6,352	69	–	–	Reverse
ND5	CDS	6,355	8,088	1,734	ATT	TAA	Reverse
tRNA-His	tRNA	8,088	8,155	68	–	–	Reverse
ND4	CDS	8,156	9,476	1,321	ATA	T	Reverse
ND4L	CDS	9,488	9,781	294	ATG	TAA	Reverse
tRNA-Thr	tRNA	9,783	9,848	66	–	–	Forward
tRNA-Pro	tRNA	9,848	9,915	68	–	–	Reverse
ND6	CDS	9,918	10,445	528	ATA	TAA	Forward
CYTB	CDS	10,445	11,581	1,137	ATG	TAA	Forward
tRNA-Ser	tRNA	11,600	11,669	70	–	–	Forward
ND1	CDS	11,686	12,636	951	TTG	TAG	Reverse
tRNA-Leu	tRNA	12,636	12,704	69	–	–	Reverse
16S rRNA	rRNA	12,681	13,997	1,317	–	–	Reverse
tRNA-Val	tRNA	14,024	14,095	72	–	–	Reverse
12S rRNA	rRNA	14,095	14,884	790	–	–	Reverse

^*a*^ –, not applicable.

## Data Availability

The complete mitochondrial genome sequence of *Rhaphidophora duxiu* is available in GenBank under accession number PP953500. The associated BioProject, SRA, and BioSample numbers are PRJNA1148785, SRS22390203, and SAMN43221142, respectively. The mitochondrial genome referenced in the text is *Rhaphidophora quadrispina.* GenBank accession number NC067624.
